# Medical and Psychology Student’s Experiences in Learning Mindfulness: Benefits, Paradoxes, and Pitfalls

**DOI:** 10.1007/s12671-016-0521-0

**Published:** 2016-04-06

**Authors:** Ida Solhaug, Thor E. Eriksen, Michael de Vibe, Hanne Haavind, Oddgeir Friborg, Tore Sørlie, Jan H. Rosenvinge

**Affiliations:** Department of Psychology, Faculty of Health Sciences, The Arctic University of Norway, N-9037 Tromsø, Norway; Department of Philosophy, Faculty of Humanities, The Arctic University of Norway, N-9037 Tromsø, Norway; Norwegian Knowledge Centre for the Health Services, N-0130 Oslo, Norway; Centre for Women’s and Gender Research, The Arctic University of Norway, N-9037 Tromsø, Norway; Institute of Clinical Medicine, Faculty of Health Sciences, The Arctic University of Norway, N-9037 Tromsø, Norway; Department of General Psychiatry, University Hospital of Northern Norway, N-9291 Tromsø, Norway

**Keywords:** Mindfulness, Stress reduction, Interpretative phenomenological analysis, Health care education, Burnout prevention

## Abstract

Mindfulness has attracted increased interest in the field of health professionals’ education due to its proposed double benefit of providing self-help strategies to counter stress and burnout symptoms and cultivating attitudes central to the role of professional helpers. The current study explored the experiential aspects of learning mindfulness. Specifically, we explored how first-year medical and psychology students experienced and conceptualized mindfulness upon completion of a 7-week mindfulness-based stress reduction program. Twenty-two students participated in either two focus group interviews or ten in-depth interviews, and we performed an interpretive phenomenological analysis of the interview transcripts. All students reported increased attention and awareness of psychological and bodily phenomena. The majority also reported a shift in their attitudes towards their experiences in terms of decreased reactivity, increased curiosity, affect tolerance, patience and self-acceptance, and improved relational qualities. The experience of mindfulness was mediated by subjective intention and the interpretation of mindfulness training. The attentional elements of mindfulness were easier to grasp than the attitudinal ones, in particular with respect to the complex and inherently paradoxical elements of non-striving and radical acceptance. Some participants considered mindfulness as a means to more efficient instrumental task-oriented coping, whilst others reported increased sensitivity and tolerance towards their own state of mind. A broader range of program benefits appeared dependent upon embracing the paradoxes and integrating attitudinal elements in practising mindfulness. Ways in which culture and context may influence the experiences in learning mindfulness are discussed along with practical, conceptual, and research implications.

**Electronic supplementary material:**

The online version of this article (doi:10.1007/s12671-016-0521-0) contains supplementary material, which is available to authorized users.

## Introduction

Caring for patients can be both rewarding and demanding. The rewarding features include mastering the skills required in one’s profession and accomplishing one’s commitment to helping patients. The demanding and stress-inducing features include containing patients’ pains and burdens, coping with treatment failures, and meeting one’s own expectations and those of others to ensure efficiency and productivity in clinical work (Wallace et al. 2009). From early on, medical and psychology students are exposed to stressors as a consequence of such demanding features, the task of developing a professional identity as well as academic achievement worries (Dyrbye et al. 2005; Truell 2001). As such stressors may threaten well-being and quality of work (Dyrbye et al. 2005; Tyssen et al. 2009; West et al. 2006), the learning of efficient stress coping stands out as a critical factor in professional development (Dyrbye et al. 2006).

Mindfulness training is an arena for stress coping. Accumulating research indicating positive outcomes of such training for healthcare professionals and trainees has increased health educators’ interest including such training in the professional curricula (Shapiro et al. 2000). Hence, systematic reviews (Irving et al. 2009; Regehr et al. 2013; Regehr et al. 2014) show that mindfulness practice is beneficial to improve health professionals’ and trainees’ quality of life, and to reduce levels of subjective stress, burnout, anxiety, and depression. Such practice may also enhance self-compassion and care, supportive listening, and empathic understanding (Chiesa and Serretti 2009; Davis and Hayes 2011; Raab 2014). These benefits may promote the quality of the therapeutic alliance, which has been found to strongly impact treatment effectiveness (Lambert and Barley 2001) and hence, success as a professional helper. Studies indicate that such benefits can be found among students in medicine (Astin 1997; Jain et al. 2007; Rosenzweig et al. 2003; Shapiro et al. 1998; Warnecke et al. 2011) and psychology (Collard et al. 2008; Shapiro et al. 2007). Interestingly, patients respond better to treatment when counselling students have been trained in mindfulness practice (Grepmair et al. 2007).

The concept of mindfulness stems from contemplative Buddhist traditions (Shonin et al. 2015). Its translation into evidence-based treatment models has, however, led to pluralism in definitions. An oft-cited definition describes mindfulness as an awareness that emerges through paying attention on purpose, in the present moment, and nonjudgmentally to the unfolding of experience from moment to moment (Kabat-Zinn 2003). Intention, attention, and attitude have been identified as core elements of this definition (Shapiro et al. 2006). Intention refers to one’s reason for practising mindfulness. Attention refers to one’s ability to focus on the present moment, as opposed to being trapped in thought. Attitude refers to the manner of one’s attention including qualities such as curiosity, openness, and acceptance. As process variables, these core elements may evolve continuously with mindfulness practice.

Amidst the popularity of mindfulness, and the enthusiasm for exploring its potential benefits, quantitative studies have focused mainly on outcome or effects. Less attention has been paid to exploring the process variables, notably intentions and the attitudes involved (Irving et al. 2014) through the use of qualitative data. Moreover, such data are highly suited to detect nuances and experiential aspects that may promote a more thorough understanding of inherent paradoxical elements of mindfulness training (i.e. *training* to promote the art of *non-striving* and the challenge of radical acceptance of momentary inner experience).

Good reasons to explore nuances and experiential aspects come from variations in the beneficial outcomes (Lomas et al. 2014; Morgan et al. 2015). Such variations are due to individual differences in perfectionism, self-criticism, and striving in the process of learning meditation (de Zoysa et al. 2014; Irving et al. 2014), in maintaining formal practice (de Zoysa et al. 2014), and coping with the increased sensitivity to ones’ own and others suffering (Christopher et al. 2011; Cigolla and Brown 2011; Keane 2014). A randomised controlled trial (RCT) of mindfulness training for medical and psychology students from our group also detected variation in overall benefits (de Vibe et al. 2013, 2015; Halland et al. 2015) in the sense that the intervention was most helpful for women and students high on the personality traits neuroticism and conscientiousness.

In the pursuit of improving the process and outcome of mindfulness training and practice, the attitude component in practising mindfulness has been suggested as a key factor in overcoming challenges (Keane 2014). Moreover, there has been a call for qualitative research examining the role of participants’ intentions in experiencing and engaging in mindfulness (Morgan et al. 2015; Wyatt et al. 2014).

The aim of this study is to examine the nature of the variation in subjective experiences of having participated in a mindfulness program, using the process elements suggested by Shapiro et al. (2006) as the conceptual framework, i.e. intention, attention, and attitude. Specifically, we focused on three related issues: (1) how psychology and medical students understand concepts and purposes of mindfulness practice, (2) how this understanding contributes to the process of learning mindfulness, and (3) the meaning students ascribe to mindfulness with regard to intra- or interpersonal life domains relevant for becoming healthcare professionals.

## Method

### Participants

Participants were recruited from the pool of students (*n* = 144) who had been randomised to an abridged 7-week mindfulness-based stress reduction (MBSR) condition in our RCT (de Vibe et al. 2013), taking place at two Norwegian universities and designed to assess how the MBSR training affected mental health. In the present, qualitative study, 11 medical and 11 psychology 1st- and 2nd-year students took part. Given the high admission grades for medicine and clinical psychology training, all participants were high achievers. Except one from Asia and Eastern Europe, respectively, all were of Norwegian origin. All had attended ≥5 of the seven sessions except two students who had attended ≤4. The mean age was 25 years (mode 21 years; two participants aged 45 years). All participating students were rewarded €24.

### Procedure

In order to optimize accessibility and acceptability, we interviewed medical students during the design phase of the project, revealing that the time commitment of the original MBSR program was perceived too extensive due to busy schedules, hence representing a barrier to participation. Since a review by (Carmody and Baer 2009) indicated that shortened versions of the original MBSR program (Kabat-Zinn 2005) may be comparably effective, we modified the program by reducing the number of weekly sessions from eight (2.5 h each) to six (1.5 h each) and the daily home practice from 45 to 20–30 min, but retained a ‘whole-day’ (6-h) session in week 7. Apart from that, our program resembles the original MBSR program by its focus on enhancing mindfulness through exercises in class and at home, as well as teachings and group dialogues on stress management, mindfulness, and mindful communication. The intervention is described in detail in the electronic supplementary material.

We conducted both individual and group interviews to obtain adequate variety, richness, breadth, and depth in the collected data (Morrow 2005). Trial interviews were conducted one week prior to the intervention to ground the study in the students’ culture and context and build sufficient trust and rapport.

Prior to the first session, students received an e-mail invitation to participate in an individual interview scheduled to take place one month subsequent to the final program session. The first author interviewed ten students (four men; three psychology students) at the University of Tromsø, and each interview lasted about 1–1.5 h. After the final program session, the participants also received an e-mail invitation to take part in focus group interviews. Twelve students agreed to participate (two men; four medical students). The first and second authors conducted two focus group interviews at the University of Oslo, each lasting approximately 2 h and comprising five and seven students, respectively.

The individual interviews were semistructured, and the initial questions focused on everyday life, probing for rich descriptions regarding social relationships, self-understanding, self-care and stress management in students’ activities and private lives (i.e. ‘can you describe a recent stressful episode and how you dealt with it?’, and ‘in which ways do you usually take care of yourself?’). Most students spontaneously introduced mindfulness when elaborating on these topics, allowing for exploration of how they related mindfulness and course attendance to different domains of their lives. Subsequently, direct questions were asked concerning mindfulness and the intervention (i.e. ‘based on your experience: what is mindfulness?’, ‘what do you experience to be the goal of practicing mindfulness?’, ‘how has it been for you to attend the course and to do the exercises?’ and ‘have you experienced any changes in your life during or after the course, i.e. in how you relate to yourself or others, or deal with stressful situations?’). The focus group interviews focused primarily on these set of issues and involved an open invitation to describe their understanding and experience of mindfulness training and comment on what others shared. When necessary, we prompted participants to enrich their narratives by exploring specific experiences and events that had been felt especially important. Being aware of the risk of positive biases, we probed explicitly for mixed, ambiguous, or negative responses (i.e. ‘which challenges did you experience in learning mindfulness?’, ‘did you experience any drawbacks or limitations with mindfulness-based stress reduction?’).

All interviews were audio recorded and transcribed verbatim by a professional transcriber and student assistants, and all identifying information was removed from the transcripts. In the presenting of results, quotes derived from the transcripts are displayed with ellipses … and square brackets […] to indicate pauses in speech and material omitted for clarity, respectively. Process and field notes were taken throughout to facilitate the recognition and acknowledgment of the interviewers’ reactions, thoughts, and observations.

### Data Analyses

The complex and ambiguous concept of mindfulness is rather foreign to members of Western cultures, and verbalizing experiences with mindfulness practices can be difficult. As experts, we aimed to identify the ambiguities in our own understanding by paying attention to tensions and doubts in the content of participant interviews (Smith 1996). Two contextual concerns were important when considering the analytical approach: the growing public interest in mindfulness, and the fact that the interviewer in the individual interviews was one of the MBSR program instructors. Considering our position as active in both contexts, we wished to explore the participants’ experiences framing the narrow program focus within a wider cultural and sociological setting. Therefore, the analyses were conducted within a constructivist-interpretivist paradigm (Ponterotto 2005) using interpretative phenomenological analysis (IPA) (Smith 2003). This involved the double hermeneutic of participants attempting to make sense of their experiences and the researchers seeking to understand the ways in which they did so (Smith and Osborn 2008). IPA aims to explore the quality and texture of individual experience, which is conceived of as uniquely embodied, perspectival and situated (Smith 1996). At the same time, IPA recognizes how such an exploration always implicates the researchers’ interpretative engagement with texts and transcripts. While examining unique descriptions of subjective accounts IPA permits the enrichment of interpretation by considering how cultural context provide a language for expressing and interpreting experiences (Willig 2001).

We found three researchers’ (IS, a female psychologist, TEE, a male philosopher, and MdV, a male medical doctor) familiarity with awareness training and meditation beneficial to the process of generating research questions and analysing the data. The remaining authors consisted of one woman and three men who were researchers from different areas of expertise in psychology and psychiatry and had extensive experience in qualitative research methods, clinical work, and student teaching. To strengthen the reflective interpretation process, these authors critically examined potentially tacitly accepted assumptions regarding the nature of the interview texts and helped to build a stepwise conceptual model consisting of both common features and individual differences. This collaborative work facilitated reflection on biases like considering some reports positive rather than mixed and, on the other hand, neglecting benefits by being too concerned with the tensions and challenges involved in learning mindfulness.

In keeping with the IPA procedure (Smith and Osborn 2008), the first and second authors carefully and independently read the entirety of transcripts from both focus groups and three of the individual interviews. They noted points and meaning in the texts and preliminary ideas for interpretation. They then reread transcripts and transformed initial ideas and notes into themes at a slightly higher level of abstraction. These themes were compared and discussed across interviews to identify divergence and common ideas with the purpose of identifying how different aspects of experiences could be grouped into broader themes and connected to overarching concepts. In a third analytical step, the themes and preliminary modelling were validated and adjusted by examining the remaining seven individual interviews, in the same way, recognizing data convergences and divergences. A third author triangulated the analysis by reading some of the individual interviews and participating in the discussion concerning emerging themes and possible conceptual models. The final organization of themes was the result of work in concert with all the researchers. Repetitiveness of themes became apparent in the last interviews, indicating an appropriate saturation point (Morse 1995), and hence, no further interviews were scheduled. To enhance trustworthiness and minimize bias, all the participants were invited to provide feedback on a complete draft of the article. They reported that the themes resonated with their experiences and suggested no critical revision.

## Results

Two overarching themes emerged. ‘Understanding mindfulness’ included the way in which students conceptualized mindfulness and their intentions in learning mindfulness. ‘Engaging with mindfulness’ involved experiences related to the journey of learning mindfulness. These themes were dynamically interrelated. Thus, conceptualizations of mindfulness influenced the way in which students engaged in and experienced mindfulness practice and integrated it into their lives, and the process of experiencing mindfulness informed and shaped students’ conceptualization as well. ‘Understanding mindfulness’ is presented below, followed by ‘engaging with mindfulness’, as a detailed illustration and elaboration according to four subcategories (i.e. ‘relaxation and concentration’, ‘containing difficult thoughts and emotions’, ‘self-acceptance’, and ‘broadening perspectives in interpersonal relationships’).

### Understanding Mindfulness

When students described how they understood mindfulness, they also highlighted their intention in practising it. Their understanding and intention often overlapped, which may reflect the fact that mindfulness is often defined both as a means and as an end in itself. Hence, they perceived mindfulness as a state of mind (‘I am just here. After a while, a sense of happiness arises’), a personal trait that can be enhanced (‘I have become much calmer’), a mode of processing (‘to view it from a distance and understand that this is only thoughts, that I can choose how to react to them’), and method (‘it’s a technique’).

Some students framed mindfulness within broad, far-reaching terms. One student described mindfulness as a way of life (‘I started noticing that this course teaches you the art of living’). Another student explained the multitude of possible intentions and ways of understanding mindfulness as follows:

Kenneth: The intention in mindfulness is pretty individual. Everybody has different thoughts on what it is. There is not one single way of defining mindfulness, and therefore, the intention, the meaning, will vary.

Taking all cases together, we identified three subcategories that covered the variations in the students’ conceptualization of their initial as well as subsequent intentions in mindfulness training. The first category was characterized by instrumentality (i.e. aiming to reach specific goals). Here, the students understood mindfulness as a technique used to achieve the desired state, such as relaxation, calmness, or self-confidence, or a tool with which to improve concentration, attention, and performance. The attentional dimensions of mindfulness (i.e. concentration, present-moment awareness) were emphasized whereas the attitudinal ones (i.e. acceptance, non-striving, non-judgementality, equanimity, kindness) were more absent.

Russ: The goal is to develop personal abilities, become better at handling stress, studies, and job situations. And a bit like mental training that I have been doing in biathlon, to be present, focus on your tasks and do things right.

Julia: What I got out of the mindfulness course was the breathing exercises. Not necessarily the mental, in the head, the thoughts, but that you calm your body. You breathe out and in deeply, and then you relax all joints and muscles. It is a technique that we have learned and can use.

The second category involved the recognition of ambiguity, identification of doubts, and exploration of tensions. The attitudinal dimension in mindfulness seemed harder to grasp than the attentional ones. Thus, even while acknowledging ideas of non-striving and acceptance as central to the mindfulness approach, such ideas seemed to compete with more instrumental aims of improvement and control. This introduced some confusion and understanding emerged as an unsettled issue. The ambiguity could have been partially due to students combining their conceptualization of the method (‘stay focused’) and aims (‘become aware of unhelpful thoughts’). Such ambiguity is illustrated in the following quotations:

Victoria: Mindfulness is about being totally present in the situation no matter what you do. To focus and not to let yourself be disturbed. To push away thoughts if they come, or not push away but accept them. […] Mindfulness is about doing things better. Well, not like performance pressure, but not to become stressed, for example.

Emma: The goal of mindfulness is getting better experiences. At least when it comes to external matters, perhaps, or I don’t know … but internal attention, that you might learn to control your breath in a way and maybe not control your thoughts but be more aware of what you think and maybe why.

The third subcategory within the mode of understanding was a more comprehensive conceptualization of intention in mindfulness training, which seemed more coherent and included several non-competing components such as self-insight and self-care, non-reactive awareness, and improved listening ability. Mindfulness was perceived as a co-arising of attentional and attitudinal qualities.

Kathy: [The goal of mindfulness is] just to know yourself better in a way. To realize that I have spun myself into a thought roundabout and manage to see this. Instead of being caught in it, feel how it feels and what limits to set. And to me, it has especially been to be good at listening. I have started to notice, during conversations with people, that so much that is exciting emerges if you only give people space instead of interrupting.

Erin: With mindfulness, it gets easier to handle emotions. Not that they are gone, but they become less pervasive, it becomes easier to contain them. And easier to contain myself too in addition to the feelings. And even to contain others and accept their opinions […] You feel so much closer to and present with others, not lost in future or past. It gets easier to sense what they feel.

The range of ways in which mindfulness were understood attracted our attention to the formative role of language upon experience. Students who reported a more comprehensive understanding of mindfulness seemed to be more engaged in practises and to perceive a broader range of program benefits. Conversely, students who took a more instrumental approach to reaching specific goals, such as improved relaxation and concentration, appeared to experience a more limited range and nature of benefits, and reported less engagement in the practices. Both the instrumental and ambiguous positions illustrate inherent paradoxes and complexities in mindfulness practices, but also the possible influence of current cultural discourses valuing performance and symptom reduction. In sum, the understanding of mindfulness and the intention and attitude in practising mindfulness appeared to shape the experience of mindfulness practice in significant ways.

### Engaging with Mindfulness

Participants’ stories revealed several domains of experience in which the process of learning mindfulness unfolded. The relationships between understanding, intention, and experience in mindfulness are elucidated throughout the subcategories.

#### Relaxation and Concentration

All of the students associated mindfulness training with the experience of ‘getting in touch with the body’. Increased bodily awareness could give rise to feelings of relaxation and calmness. Students also reported becoming aware of their inner judgement and rumination, the extent to which their minds wandered, and how strongly thought processes contributed to negative cycles of stress reactivity. They ascribed the experience of relaxation caused by a shift in attention from immersion in mental activity to becoming increasingly focused on sensory perception.

However, some students struggled with doubt, impatience, or boredom during practice. When this was the case, they felt as though they had failed or the exercise had not worked. Others realized that the origin of their struggle was their agenda in wanting to achieve a certain outcome (e.g. a relaxed state), as Susan explained:

I found that being right here and now and not thinking so much led to relaxation, even if it wasn’t a goal, it happened several times. Because it happened, I started to try to achieve it, and when it became a goal, it became more difficult just to relax and be present here and now. Actually, it was like this quite often for me, meditation was pretty straightforward, but I managed to make it difficult.

A few students explicitly established the ability to concentrate and increase performance as their personal goal in the training and tailored their practice accordingly:

Carla: I have not been sitting there for 5 or 10 minutes because I get impatient, I get like “no, you know what, this is not effective!” Instead, I use it to bring myself back, to manage to stay focused. I can lie down and feel how it is to be here right now and all that stuff, but then you don’t really *do* anything. It gets very far out relative to real life to me.

The last part of this citation indicates waning motivation for lengthy, formal mindfulness practises, which did not match the participant’s motivation for achieving a fixed goal, thus inhibiting the opportunity to overcome common initial barriers and difficulties with practise. Similarly, some felt so in control of the situation, both socially and educationally, that they were less motivated in further post-program practise. Russ elaborated on this:

I have not done many exercises since the course. When I must concentrate on something I bring myself back to focus quicker than before, but apart from that, I have not felt any need to use it yet. When I am not stressed, it is harder to do, and I am not sure what I get out of it. Then it is actually harder to practise. However, I like to think that I have it as a tool when the time comes.

Some participants, who initially just aimed to improve their concentration or learn about a tool for relaxation, seemed to transcend these objectives as part of the process of becoming increasingly aware of their mental habits. Such increased awareness appeared to facilitate spontaneous questioning of the validity of thoughts and beliefs. Ultimately, Carla also recognized this:

It was kind of strange. I have started to notice what I actually think about. I have really never done that before. I think “Where are my thoughts now and why are they there?” in a way and fix what made them come in the first place. Give myself some answers, to put it that way, so that I maybe do not think about it that often. Then it is easier to return to the present moment again.

For the students who noticed and reflected on the content of their thoughts and beliefs in this way, mindfulness seemed to facilitate cognitive reappraisal of stressful situations, which yielded more insight into stress reactivity processes and negative cycles.

#### Containing Difficult Thoughts and Emotions

Many students reported that, as they practised, they began to observe thoughts and feelings from a new perspective, which was described by one student as ‘going meta on myself’. From this perspective, thought content and emotions were experienced as less overwhelming and allowed students to react with more flexibility rather than becoming caught up in them.

Mark: To find out, it is very cool, just discovering how emotions change as you have different thoughts, how incredibly changing it can be! When you notice that, it turns thoughts into clouds in a way, in consciousness. It’s just thought patterns. I think it is good that you render them harmless.

Jessica: You manage to take a step aside and lift yourself above a bit and take a look at it from a distance and see that it is not necessary to be stressed because this is only thoughts. I can choose how to react to them in a way.

At times, practising mindfulness exercises gives rise to negative feelings such as anxiety, anger, or sadness. Kenneth had experienced the sudden death of a patient at work; this was a traumatic event that entered his consciousness during a mindfulness exercise in class. It evoked shame and guilt: ‘You calm yourself down to the degree that you actually can think things through. Both good and bad stuff can bubble up to the surface’. The instructors guided him in approaching his feelings more openly and non-judgmentally. Allowing this very emotionally loaded memory to remain in his consciousness helped him to extend the view of his role and reduce self-blame. However, at home after the class, he again experienced the return of the unpleasant memory and used mindful breathing as a means of avoiding it, which contributed to doubt and confusion regarding mindfulness as a useful coping method.

I maybe feel that I at times run away because I choose to think about the breath only and concentrate on the breath instead of dealing with the problem, in a way. I am maybe the type who wants to fix it you know, instead of just sitting and looking at it, in a way, and “doing nothing”, then I want to fix it.

The understanding and experience of mindfulness was ambiguous: the instruction to ‘just sit and look’ was associated with passivity and contrasted to the notion of actively controlling or ‘dealing with’ discomfort. Exploring mindfulness (of breathing) more instrumentally as a distraction technique temporarily increased the sense of control over discomforting emotions, but was felt as an act of avoidance. Thus, tolerating strong negative emotions was challenging and appeared to require active guidance and support. However, the recognition, in class, that strong emotions are tolerable promoted the practice of observing of one’s discomfort more openly and curiously rather than avoiding it, thereby lowering the need to change it. This represented an active and more functional cognitive-emotional coping approach. Thus, even if the understanding and experience of mindfulness was ambiguous, Kenneth expressed that some reconciliation occurred, and he later applied for a job in the workplace that he had avoided since the incident.

Rather than trying to fix or avoid strong negative emotions, some students reported that just being aware of emotions could have the paradoxical effect of reducing their intensity. Alan described the person renting his room moving out without paying his rent. Acknowledging his anger made it possible to contain it:

I literally felt the anger boil inside me. Then I thought, “OK, now I’ll be conscious of what I feel, now I am angry, right” and then “OK”, you know, and then in a way, it cooled off a little when I became aware of the feelings, then it became a shifting, you know, like anger… awareness… anger… awareness. While before I would probably waste two hours being pissed off and go to bed exhausted.

Similarly, Erin described how her relationship with anger had changed with greater awareness:

I experienced a big change during this semester, in that I didn’t actually identify with the anger but instead reflected on what happened inside me. Then I managed to change it; so now, if I get irritated, it’s not a big explosion, in a way. It’s more like I can live with the feeling, but it isn’t that overwhelming. I have become much calmer.

Performing the exercises helped participants to step back from the stream of thoughts or emotions that occurred, which made them more tangible and manageable.

#### Self-Acceptance

Self-acceptance was a frequent implicit and explicit theme in the training. Some students felt that they mastered everyday life well. To them, addressing the concept of self-acceptance directly in class appeared irrelevant, superfluous, or diffuse. One student said that she was surprised when the instructor introduced the concept of acceptance, because she could not identify anything that she needed to accept. In contrast, there were several students who described the social context of being a medical or psychology student as demanding. They felt that cultural and subcultural ideals and norms made it difficult to navigate and listen to themselves on a day-to-day basis. Johanna illustrates this:

Those who are perfectly dressed with perfect makeup at 7.30, they set a certain standard. Clearly, when everybody appears as if they master all the demands that seem unreasonably high to you, it is hard to see that they are unreasonably high because it seems like everybody makes it.

Several students reported relating to themselves rather critically. For some, taking part in the program led them to discover how pervasive this self-critical stance was. Johanna stated:

I actually became aware of the fact that it is a process, that I judge myself. I have never thought about it before, that you create the bad feelings yourself. I need to be kinder to myself. I don’t exactly know how to do that, but I often put far too high demands on myself. I would never act like that towards anybody else.

The need to be kinder to oneself and more patient with one’s inner experiences was recognized, but the process of increased acceptance and self-care was part of ongoing exploration. Despite being an appealing concept to many, their stories strongly suggested that self-acceptance was harder to integrate into their lives. Emma expounded this:

I think it’s a good idea really, just accepting things as they are. It is easy to say to oneself, “Just accept how it is.” However, at the same time, there is something inside me that says, “You have to improve.” In the end, you don’t bother to say “accept” anymore! You have to say it and believe in it at the same time. You must take it all the way inside you in a way.

As reported by several participants, time pressure and stress may be barriers to this process, decreasing engagement in practises. Others, such as Mark, practised mindfulness exercises every day, both during and subsequent to the program. Mark had recently overcome a period of depression and described himself as quite self-critical. He associated the practice of mindfulness with non-striving but also considered it a possible avenue to changing himself as a person.

I really think about it a lot, I want to be very yogi-like. They seem so calm and centred. You just relax, move, float. […] I want to be more in the present moment; that’s what it is about. The techniques for being in the now, even if you never in a way become Mr. Mindful.

Mark experienced a certain tension between desired and actual psychological reality and described conflict between striving and the idea of non-striving when practising mindfulness:

I just doubt myself, whether I am doing the practices right, so to speak. Because sometimes it feels so good, but the next time it maybe doesn’t feel that good. […] Then I think, “You are not trying to achieve anything.” Whether it is good or bad, tense or relaxed, it means nothing. That is a comforting thought, I think. Then you can reject mistakes right away.

Practising mindfulness reminded him about the impossibility of ‘becoming Mr Mindful’ and the choice to let go of striving to achieve an ideal state or self. Some students clearly appeared to experience mindfulness training as a means of fostering greater self-acceptance. These students were usually relatively stable and engaged in mindfulness practices during and subsequent to the program. Some were also familiar with yoga, meditation, or ideas originating from contemplative traditions. Alan had practised meditation prior to joining the program but explained how his intention in meditation changed during the mindfulness classes:

We all have that mud, and we just have to live with it. We will never fix it! Previously, I really wanted to initiate a project (with meditation) to get rid of the problems once and for all, like a Marathon! Now, I believe the problems must be there, so I laugh if people say, “Ah if I do this I will no longer feel fear and never be sad or afraid again!” I used to be like that; I used to think like that.

Gradually, mindfulness was experienced as a life-long journey involving courage and the act of embracing imperfection and the suffering that life entails.

Jason: It means something to me to dare to listen to oneself, and you are almost forced to listen if you increase your focus on the body, which was most important to me. You learn to listen to yourself in a way, and that will lead to a kind of self-care.

Linda: To me, mindfulness was a channel for learning to take a little bit more care of myself. To learn to meet myself with the generosity, care, and kindness with which you naturally embrace others. […] To live in a constant ought-to mode is distressing to the body in a way, and when you practise mindfulness, you get a break from this ought-to mode.

Students who experienced mindfulness training as an avenue to better self-care and compassion could detail the ways in which they coped with the performance pressures and demands imposed by themselves or others. Nevertheless, some conceived mindfulness as a somewhat limited version of ‘time-out’, yet no real alternative to the usual ‘ought-to mode’ dominating their approach to everyday life.

#### Broadening the Perspective—Relationships with Others

For some, performing mindfulness exercises also improved the quality of their interpersonal relationships. These students talked about experiencing greater patience when interacting with children, partners, friends, or strangers. In particular, they resisted the temptation to interrupt or try to ‘get somewhere’ in a conversation, started to listen more carefully to peoples’ stories, and paid attention to how they reacted to these stories instead. Increased attention to oneself was reported to strengthen their listening ability. Jason described this as follows:

The fact that I focus on myself, I feel, makes me better at listening to others. I feel that when I am listening to people, I think, “Now I will be aware of my own body” more and it actually makes me better at listening.

Few participants had contact with patients during the study. However, some described how increased presence affected patient contact. Kenneth stated the following:

I notice that I remember things that patients tell me much more than I did earlier. It is as if I didn’t listen before. Now I know more, I remember more of their stories. I have taken more time at that moment with that patient.

Being mindful of bodily sensations helped students to remain grounded and present, both with the other person and their own reactions rather than neglecting or being overwhelmed by challenging emotions. Jessica explained how mindfulness practices helped her to contain her own reactions when meeting patients experiencing intense suffering:

A patient who had a really tough time told her story, and if I had watched her on TV, I might have started to cry, you know. But when you can’t cry then you must have another way. For example, feeling the contact with the ground, breathing. I use the breath as a kind of anchor so that I don’t get totally blown away.

Similarly, Linda described an episode on the train when a beer-drinking stranger in dirty, bloodstained clothes took a seat in front of her. She felt disgusted, irritated, and anxious. She then described establishing contact with her breath and experiencing a shift in orientation towards the stranger:

But then I just started to breathe a bit and relax and feel where I was, and like, “yes, OK, here I sit and there another person sits, in another place.” He had obviously had a tough night. […] Just breathing and practising being present helped me accept the way things were there and then.

The act of becoming increasingly aware of the moment-to-moment experience appeared to facilitate her acceptance of the unpleasant situation and increased her compassion for the other person.

## Discussion

Our findings indicate that students’ mode of understanding and their personalized intentions in learning mindfulness were dynamically related to their actual experiences and the extent of their engagement in mindfulness training*.* The understanding ranged from ‘instrumental’ to more ‘comprehensive’ positions, and correspondingly, the process of engagement varied from reaching a specific state of mind to broadening the perspective in relation to self and others. Different positions of understanding were neither static nor mutually exclusive but could be drawn on simultaneously; however, more complex and ‘comprehensive’ conceptions of mindfulness often seemed to be associated with a larger actual engagement in practices. As a follow-up of the RCT study, the present study enriched the overall picture of program benefits by identifying diversity, tensions, and paradoxes in the ways in which mindfulness is understood and experienced. Findings attract attention to the importance of intention and attitude in the process of engaging in mindfulness training.

Most students experienced mindfulness as an avenue to listening more attentively to their inner worlds of bodily sensations, needs, and stress signals, thereby promoting the reappraisal of situations and formation of new insights into intrapersonal processes. This supports the notion of mindfulness as ‘self-attunement’, i.e. a state in which one seeks to remain attentive to one’s own internal world with compassion and kindness (Siegel 2007). This broadened state of awareness, sometimes referred to as decentring (Fresco et al. 2007), reperceiving (Shapiro et al. 2006), or metacognitive awareness (Teasdale et al. 1995) implies a shift of perspective to attend to experiences in a more objective and accepting manner. This facilitates the ability to contain strong emotions and negative thought processes and respond more flexibly and adaptively to stressful situations, i.e. decreased reactivity and increased response flexibility (Davis and Hayes 2011), and more adaptive affect regulation (Williams 2010). This is an outcome of mindfulness training reiterated in both our qualitative study and our RCT (de Vibe et al. 2013). Hence, mindfulness practice may represent an opportunity for increased emotional exploration and containment, which is important for therapist empathy (Keane 2014) and therapeutic alliance (Bruce et al. 2010).

In addition, as a result of self-attunement, the participants experienced the abridged MBSR program as legitimizing self-care and self-compassion (Smeets et al. 2014) and the embracing of their imperfections and vulnerabilities. This is an important capacity, and in particular for those participants who usually attune to other people’s needs and to high social and professional demands on the expense of their own needs. Hence, for high-achieving students, MBSR may serve as buffer against the high levels of stress (Shanafelt et al. 2010). Furthermore, self-compassion has been strongly positively correlated with empathic concern (Kingsbury 2009) and the capacity to resonate and connect with others (Neff and Pommier 2013).

Students who were most deeply engaged in the training typically associated mindfulness with previously established ideas and patterns in valuing self-exploration and the dynamic interconnection between mind and body. High engagement was also reported among those who were in touch with the emotional challenges present in their lives as well as in their study-situation. Students with this deep engagement also typically expressed a more comprehensive understanding (i.e. perceiving mindfulness as a gateway to self-exploration, interpersonal attunement, and learning non-reactive meta-awareness) of mindfulness and reported richer experiences with how mindfulness skills may be transferred to several areas of life. The attentional (i.e. present moment awareness) and attitudinal process elements (i.e. acceptance, equanimity, non-striving, non-reactivity, non-judging) of mindfulness were given equal attention. Thus, a comprehensive understanding was associated with the clear intention to attend to experience with this particular set of attitudes; however, developing and maintaining these attitudinal qualities still was depicted as an ongoing process of learning from moment to moment.

By contrast, students that tended to express a more ‘instrumental’, goal-oriented understanding of mindfulness emphasised the attentional dimensions of mindfulness, and accentuated mindfulness as a way of achieving relaxation or concentration, generally associated with lower levels of engagement. The ambiguous conceptions of mindfulness reflected understanding in evolvement, challenged and developed by engagement with mindfulness practices. Here, difficulties and tensions in making sense of and developing the attitudinal qualities of mindfulness were especially pronounced. The reduced intensity and duration of the present intervention might have limited the students’ opportunities to explore mindfulness practices more fully and thus contributed to the heterogeneous experiences. However, our findings concur with a review of qualitative mindfulness studies in which the majority employed the original duration and intensity (Morgan et al. 2015), stating that health care professionals who used mindfulness as a ‘tool’ were less likely to develop a ‘deeper understanding’ of mindfulness as compared to those who adopted mindfulness as ‘a way of being’.

The experience of being in a MBSR group with fellow students was infrequently mentioned by the participants, and also revealed heterogeneity. Some experienced that the group context promoted a focus on normalisation and recognition of shared human vulnerabilities, echoing patterns in existing research (Beckman et al. 2012; Morgan et al. 2015), while others expressed less comfort with disclosing themselves freely to classmates. The younger age and higher importance of social norms regarding ‘mastery’ may be a reason for this reservation.

Our results emphasize the benefits of meeting experiences with an accepting, non-judgemental, and open attitude; however, they also highlight the complexities and challenges faced in integrating these attitudes. By its very nature, mindfulness opens up numerous paradoxes: students are urged to practise but simultaneously allow themselves to be with what is without trying to change anything (Kabat-Zinn 2003). Despite recognizing the importance of the attitudinal components of mindfulness training in experiencing health benefits (Baer et al. 2006), the challenges and difficulties involved in cultivating a spirit of non-striving, acceptance, and compassion are mostly unexplored in psychological literature (Grossman 2015). All individuals continuously judge their inner experiences, situations, and other people. Mindfulness misinterpreted as the capacity to accept all psychological experience without judgement can easily remain perceived as just another good idea or an unattainable ideal to impose upon oneself. However, in essence, mindfulness entails an invitation to metacognitively observe one’s habitual judgement non-judgmentally and acknowledge and provide space for a lack of acceptance and inner struggle, when present. Therefore, the degree to which people grasp the paradoxical nature of the mindfulness process may be one means of understanding the observed diversity of experiences with mindfulness training.

Mindfulness attitudes, involving letting go of the need to control or ‘fix’ psychological experiences, tend to be more easily remembered and integrated over time and are sustained by regular mindfulness practice (Malpass et al. 2012). Acknowledging that people approach these practices in different ways at different points in their lives, we suggest that mindfulness teachers should increase attention to and repeatedly inquire into participants’ intentions, attitudes, and interpretations of mindfulness during mindfulness-based programs. The overall purpose here is to facilitate an exploration of the inherent paradoxes of mindfulness practice, the tensions between striving and non-striving, instrumentality and non-instrumentality, and between acceptance versus the natural wish to achieve something with mindfulness training. Eventually, such inquiries may promote an understanding of the journey as the goal in itself (Bush 2011).

However, grasping the paradox of mindfulness and mindfulness training goes beyond being a didactic task as the task mirrors conceptual and cultural challenges. In our opinion, there is a gap between the original intention of mindfulness as a gateway to wisdom, insight, and compassion (de Silva 2005) and Western society’s drive for instrumental, quick-and-easy paths to self-actualization, seeking tools to bring about symptomatic relief and search for remedies for all of our ills and ailments (Rose 1990). Mindfulness has, to an increasing extent, been adopted by popular media and mainstream psychology and presented as a tool to reduce symptoms and increase health, happiness, and performance (Purser and Loy 2013) within the frame of a cultural fixation on the individual ‘unique’ self.

Fundamentally, however, mindfulness practice offers perspectives on health and well-being that depart from dominant cultural ideas of ‘feelgoodism’ and self-optimization. Rather, mindfulness involves learning to embrace uncomfortable psychological phenomena and perceived imperfections rather than trying to overcome or discard them, and is oriented towards the existential commonalities between persons, and their interdependence and interconnectedness within a larger whole (Kabat-Zinn 2003). In a cultural framework where symptomatic relief, performance, and adaption are dominating values in mental health care and public health services (Binder et al. 2010), humanistic or existential values (such as self-insight, compassion, or interconnectedness) tend to be marginalised. Moreover, as mindfulness has been popularized and legitimated largely through neuroscience and as an add-on to cognitive behavioural treatments, this may blur the social, contextual, and values-based aspects of mindfulness practice (Kirmayer 2015) as well as the original intentions of mindfulness as a gateway to insight and wisdom.

The variety in participants’ understanding of mindfulness mirrors the lack of common ground in the definitions of, and intentions in mindfulness in this complex cultural context, intervention delivery, research, and theory (Grossman 2011); however, it could also be explained by this.

In addition to confirming the centrality of the mindfulness components of attention and attitude, our data largely support Shapiro et al.’s (2006) inclusion of intention as an essential component in understanding the processes involved in mindfulness. This component has often been overlooked in other definitions of mindfulness (Bishop et al. 2004; Brown and Ryan 2003) and empirical research (Irving et al. 2014). Shapiro (1992) found that meditators’ intentions with practices shift along a self-regulation, self-exploration, ‘self-liberation’/compassion continuum. Our results show that the intervention emphasized the self-regulation (i.e. stress management, concentration) and self-exploration (i.e. insight, meta-awareness, self-acceptance) aspects of mindfulness. However, only a few of our students described the qualities of feeling more compassionate towards others or feeling more connected with nature (‘self-liberation’). In our opinion, the students’ stories demonstrate the relevance of an ongoing theoretical discussion in contemplative research concerning the ‘correct’, fruitful, or merely different intentions involved in practising mindfulness (Brito 2014; Lindahl 2015; Monteiro et al. 2015). In the context of health care education, the potential values of, as well as dilemmas in emphasizing the discourse of interdependence and compassion—with its implicit ethics—merits particular consideration.

### Limitations and Future Research

Any theoretical generalization of the results of this study should be limited to similar Western settings with the same social ideas regarding health and self-improvement. In addition, the participants were medical and psychology students in their early twenties; therefore, our findings may not be generalizable to healthcare professionals with varying durations of clinical experience. Relatively few men participated in this study, and we encourage future research on the underexplored topic of gender and subgroup variation in experiencing mindfulness. Furthermore, participants were self-selected, and their personal characteristics may differ from those of students who chose not to participate in the interviews. However, positive bias due to self-selection and the impact of the researchers’ double role as interviewer and class instructor appears to have been countered by our active search for negative or mixed responses and the fact that students participating in the in-depth interviews gave their consent to be interviewed prior to their involvement in the intervention.

The complexity of learning mindfulness practice merits the use of mixed quantitative and qualitative designs intended to explore both outcome and process variables. Through analysis of program material, taped records of sessions, and instructors conceptions of mindfulness, future research should take into account the definition and communication of the aims of mindfulness training in the particular context of intervention delivery. This kind of research may enrich our understanding of the influence of broader sociocultural conceptual frameworks on the experience and understanding of mindfulness. Moreover, mindfulness training rests on the assumption that the teachers’ own engagement with practice counters the risk of teaching ‘caricatures of mindfulness’ (Kabat-Zinn 2003), and further research focusing on the teacher-participant relationship and how this evolves during the mindfulness program may fill a gap in knowledge. In addition, in order to determine the external validity of the intervention, it would be useful to examine and compare different approaches to mindfulness training both regarding duration, intensity, and content, and examine the ways in which it could be integrated into educational contexts (Cohen and Miller 2009; Mascaro et al. 2013; Pakenham and Stafford-Brown 2013), including mindfulness programs tailored to address individual differences in participants’ intentions, understanding, and conception of mindfulness.

## Electronic supplementary material

Below is the link to the electronic supplementary material.

ESM 1 (PDF 1692 kb)
